# Green-Labelled Rice versus Conventional Rice: Perception and Emotion of Chinese Consumers Based on Review Mining

**DOI:** 10.3390/foods12010087

**Published:** 2022-12-24

**Authors:** Heng Xu, Mengyun Xiao, Jun Zeng, Huihui Hao

**Affiliations:** 1 School of Management, Henan University of Technology, Zhengzhou 450001, China; 2 Faculty of Agribusiness and Commerce, Lincoln University, Lincoln 7647, New Zealand

**Keywords:** green food, consumer perception, topic modeling, sentiment analysis

## Abstract

The COVID-19 pandemic increased public health awareness, changing consumers’ sensitivity and beliefs about food health. Food anxiety and health scares turn consumers toward safe and healthy foods to strengthen their immunity, which makes green food more popular. However, it remains unclear how to understand the gap between consumer intention to purchase green food and their actual purchasing behaviour. Taking rice as an object of study, comparing differences in consumer perceptions and emotions towards green-labelled rice and conventional rice is beneficial for understanding the components and psychological characteristics of consumer perceptions of green food. Therefore, we used topic modelling and sentiment analysis to explore consumers’ focus of attention, attitudinal preferences, and sentiment tendencies based on the review (*n* = 77,429) from JD.com. The findings revealed that (1) consumers’ concerns about green-labelled rice are increasing rapidly, and most have a positive attitude; (2) consumers of green-labelled rice are more concerned about origin, aroma, and taste than conventional rice; (3) consumers of conventional rice are more concerned about the cost-performance ratio, while consumers of green-labelled rice are also price-sensitive; (4) green label mistrust and packaging breakage during logistics are the leading causes of negative emotions among consumers of green-labelled rice. This study provides a comparative analysis of consumer perceptions and emotions between the two types of rice, thus revealing the main influencing factors of the intention-behaviour gap and providing valuable consumer insights for the promotion of green consumption and the sustainable development of the green food industry.

## 1. Introduction

The popularity of green consumption has led to a growing interest in green food [1]. In particular, the pandemic has increased public awareness of food safety and environmental awareness, and strongly influenced consumers’ habits and behaviours [2], leading to healthier lifestyles [3]. Due to personal concerns about safety and health, consumers are eager to strengthen their immunity through healthy eating [4]. With its specific attributes, green food meets the current demand [5]. Research demonstrated that consumers’ perceived severity of the epidemic and their own safety awareness contribute to their intention to purchase green food [6]. Qi et al. [5] also concluded that the outbreak of the COVID-19 pandemic has strongly influenced consumers’ habits and behaviours, creating a more sustainable and healthier era of consumption. In this context, there is a high interest in the sustainability of food choices [7,8]. However, understanding the gap between consumers’ willingness to purchase green food and their purchasing behaviour remains a pressing issue [9]. In order to identify the causes of the “intention-behaviour” gap, it is essential to explore consumers’ attitudinal preferences and emotional dispositions towards green-labelled food and conventional food.

Green food is produced in an excellent ecological environment and meets technical standards of safety, nutrition, and non-pollution [10]. Due to the strict control of the ecological environment where agricultural products are produced, green food not only prevents people from food-borne hazards caused by food contamination but also plays a crucial role in reducing carbon emissions and protecting the natural ecological environment. Besides, it is widely believed that green food is produced in an environmentally friendly way and is healthier than food produced traditionally [11].

Nearly half of the world’s population relies on rice as a staple food. Although rice grown in a green sustainable environment sells for a higher price in the global market, it is increasingly attracting consumer attention [12,13]. Moreover, people are willing to pay for rice with environmental attributes (i.e., green-labelled rice) rather than rice without environmental attributes (i.e., conventional rice) [14]. The study showed that consumer attitudes toward green-labelled rice are positively correlated with concerns about food safety and the perceived importance of environmental consequences [15]. In addition to health and environmental protection, a traceable label certification system is a significant factor in consumers’ positive attitude towards green-labelled rice, even among those with little knowledge of eco-labelling standards [13,16].

Product reviews on e-commerce platforms generally require consumers to confirm receipt before they post anonymously. These reliably reflect consumers’ authentic evaluations and opinions on the quality, price, and experience of the purchased products, providing a large amount of data to support our study of consumer attitudes and behaviour from the user perspective [17,18]. Therefore, through text mining we can understand the potential focus of consumers, explore the actual trend of consumers’ green consumption during the COVID-19 pandemic, and analyse the difference between consumers’ focus and attitude when buying green-labelled rice and conventional rice. Based on the above results, through further use of the sentiment analysis method, we can observe the changes in the emotional characteristics of consumers towards different food products [19] and analyse the thematic preferences of positive and negative emotions of consumers towards two types of rice. These results will help us to compare the differences in consumers’ perceptions and emotions towards different food products and explore consumers’ purchasing behaviour.

Taking green-labelled rice and conventional rice as the research object, this study selects user-generated content (i.e., product reviews) from JD.com in China as the data source. Then, the topic modelling and sentiment analysis are adopted to extract consumers’ attention and compare consumer attitudes and sentiments towards the two types of rice, so as to gain insight into the components and psychological characteristics of consumers’ perception of green food.

## 2. Literature Review

### 2.1. Consumer Attitude towards Green-Labelled Rice

Consumer attitudes are the main drivers behind consumption behaviour [20], and consumer attitudes towards green-labelled rice significantly influence their choices [21]. Green-labelled rice is significantly better than conventional rice in terms of nutritional quality and has fewer heavy metals than conventional rice, making it more compliant with safety and environmental standards [22]. Most studies have shown consumers’ positive attitudes towards green-labelled rice rich in vitamins and other nutrients [13]. In China, consumers have shown a favourable preference for green-labelled rice due to health benefits and environmental protection considerations [16].

Previous studies have focused on the factors influencing the purchase intention of green-labelled rice. Firstly, health and food safety are the essential attributes for consumers to choose green-labelled rice [22], as people generally believe green-labelled rice is healthier and safer than conventional rice [23]. Secondly, in the context of a low-carbon society, consumers’ subjective environmental knowledge has a significant effect on the purchase of green-labelled rice with environmental attributes [24]. This is because the production of green products is less polluting to the environment, and it is a prosocial and environmentally friendly behaviour [25]. Finally, consumer trust in green labels is an essential factor influencing consumers’ purchasing behaviour [26]. It contributes to the purchase and attitudinal loyalty of green products, and a nutritional label or brief claims will improve consumers’ attitudes towards rice [26]. Samant and Seo [27] also concluded that the effects of sustainability-related label claims on quality perception and acceptability of food become significantly more pronounced when consumers understand and trust the label claims.

Emotions are considered to be important drivers of food-related cognition and behaviour [28]. According to the technology acceptance theory, the acceptance of new technology or models demand positive attention and sufficient understanding from consumers [29]. By paying attention to the new things and perceiving their characteristics, consumers generate sentimental preference for these new things and then produce a behavioural intention [30]. The traditional approach of consumer sentiment research is mainly through verbal self-reporting questionnaires [31] which is easy to apply, cost-effective, and discriminatory, but may not capture the full range of emotions individuals might experience in response to food, and therefore may not properly measure food-evoked emotions [32,33]. Alternative methods based on big data for sentiment analysis are becoming popular [34], and sentiment classification methods using machine learning and deep learning are widely used in lots of studies. Compared to traditional self-reporting questionnaire methods, sentiment analysis, through online reviews, allows us to capture the emotions evoked by consumers’ direct experience with food [28], enabling a more objective determination of consumers’ emotions and attitudes toward green foods. It is worth noting that although some scholars have proposed the main reasons that affect consumers’ negative attitudes towards green-labelled rice as the distrust and lack of knowledge about green labels [18], no study has yet illustrated consumers’ positive and negative attitudes towards buying green-labeled rice by comparison with conventional rice.

Consumer attitudes are linked to a complex set of ideas, motivations, and experiences [35]. The sustainability of the green-labelled rice industry depends on consumers’ concerns and attitudes toward green-labelled rice [36]. Therefore, a comparative analysis of consumers’ attitudinal preferences and emotional dispositions towards the two types of rice based on user-generated content provides a new perspective for the green food sector and helps to understand the gap between consumers’ willingness to buy green food and their actual purchasing behaviour.

### 2.2. Text Mining in Green Labeled Food

Green food in China refers to a wide array of primary and processed agricultural products that are safe, nutritious and of high quality for human consumption. Hassan et al. [37] argue that the term “green food” considers not only the culture of food production, but also the protection of the environment, economics of stakeholders, and social relationships. Organic food, which also has safety and environmental attributes, is not accepted widely among the population due to the most stringent certification standards. In contrast, green food has higher consumer awareness and is widely followed in China [38], and researchers are increasingly interested in green food consumption. Therefore, this study also focuses on green food.

Most previous studies have used traditional questionnaires or interviews to explore consumers’ preferred motivations for purchasing green-labelled rice [16,39], as well as their purchase intentions and influencing factors [24]. However, regional surveys are limited to the themes covered by research questions and usually only capture small or moderate sample sizes (Table 1), making it challenging to explore the perceptions and attitudinal preferences of multi-faceted populations on health issues [40].

Considering the anonymity of the Internet, consumers tend to express their opinions and tastes freely on review sites and share genuine reviews and experiences of products [44,45]. Online reviews thus convey emotional information about consumers and reflect their attitudes towards products [46]. The data from online reviews can be mined to obtain more practical and objective information, as it is not influenced by the subjective will of the sender [43].

Text mining approaches are gaining popularity in research in the field of food science [40]. We can use the LDA model to extract the focus of consumers’ attention on green-labelled food from a large amount of review content, and sentiment analysis methods can be used to analyse consumers’ attitudinal preferences towards green-labelled food. In recent studies, Hao et al. [43] analysed the logistical factors affecting consumer satisfaction when purchasing rice and produce through text mining techniques based on consumer comments on the JD.com. Singh et al. [18] analysed public attitudes towards green-labelled foods by using thematic clustering and the sentiment lexicon approach based on Twitter comments. Thus, text mining methods can offer more objective insights into consumer attitudes towards green-labelled rice to provide deeper insights and more diverse recommendations [47], which can help promote sustainable development in the green food sector.

## 3. Methodology

**Figure 1:**
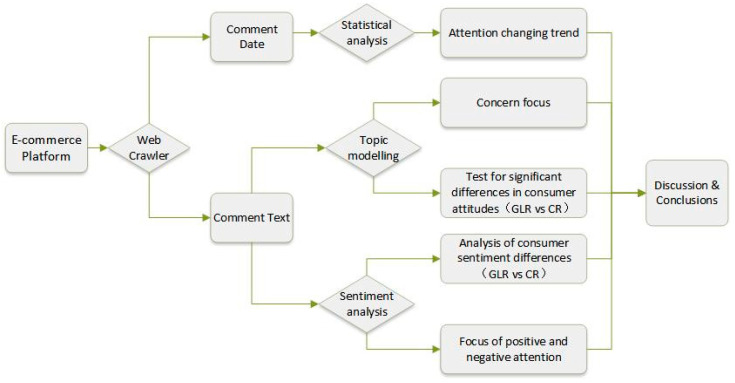
The analysis framework of review mining.

As shown in Figure 1, firstly, we used web crawling techniques to obtain consumer review data on green-labelled rice and conventional rice in JD.com and preprocessed them accordingly. Secondly, the LDA topic model was used to extract topics from the review text in order to uncover the focus of consumer attention and to conduct a significant difference analysis. Finally, the SnowNLP was used for sentiment classification to identify consumers’ sentiment tendencies and analyse the causes of different emotions through word frequency statistics.

### 3.1. Chinese Green Food Label

**Figure 2:**
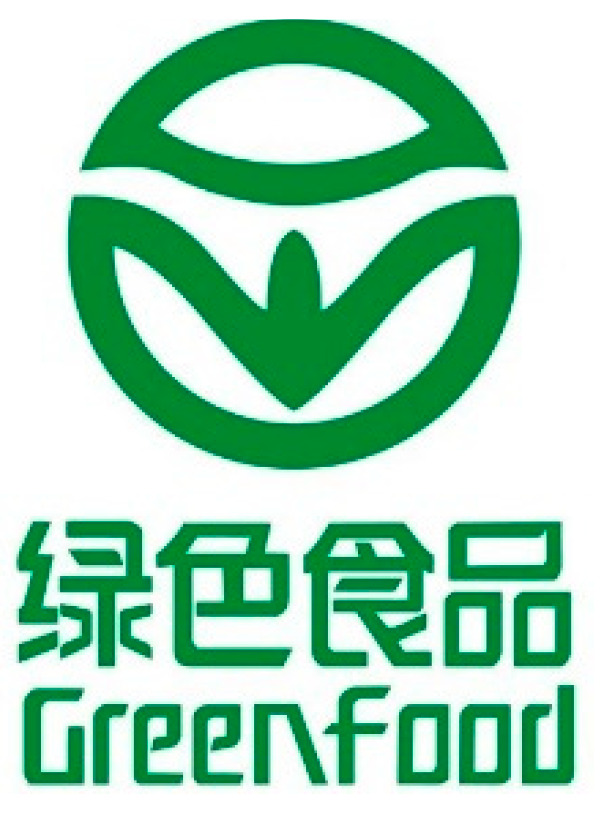
Chinese green food label.

The green food label (Figure 2.) is a product quality certification mark officially registered by the Green Food Development Center of China with the Trademark Office of the State Administration for Industry and Commerce. Green food regulations and standards were developed and established following the Codex Alimentarius programmed by the Codex Alimentarius Commission (CAC) [38], which is a unique labelling certification in China [16]. It consists of three parts: the sun above; the leaf below and the bud in the center; and the logo is a square circle, meaning protection and safety. The logo indicates that the environmental quality of the place where the product is made conforms to the Environmental Quality Standards for Green Food Production Areas. The production process is strictly under the guidelines for the use of green food production materials and the requirements of the production operation procedures, meeting the technical standards of safety, nutrition, and non-pollution. The logo is awarded by the Green Food Development Center and the Green Food Certification Review Committee after a systematic assessment and certification. Green food follows a “from farm to fork” control principle similar to the Hazard Analysis Critical Control Point (HACCP) system [38].

### 3.2. Data Collection

This study chose JD.com as our data source. JD.com is one of the most popular B2C platforms in China’s e-commerce sector, with 570 million active annual users and a well-established consumer post-purchase evaluation mechanism [48]. According to the sales ranking of the products on JD.com and the fan base of the store where the product belongs, the rice brands that are well-known and popular among consumers were selected as follows: October Paddy (fans: 15,168,000), San Ren Xing (fans: 1,226,000), Tian Yuan Dao (fans: 2,159,000), Chai Huo Da Yuan (fans: 10,107,000), Qiao Fu Da Yuan (fans: 299,000), and National Treasure Qiaomi (fans: 1,050,000). In addition, the number of product comments of these brands is over 100,000, providing us with rich data from which to extract valuable information using text mining methods [49].

In April 2022, we used the Python crawler to obtain product reviews for the above brands of green-labelled rice and conventional rice products, including user ID, comment posting times, and comment texts. A total of 82,398 raw comments were obtained, of which 41,394 were for green-labelled rice and 41,004 were for conventional rice. Furthermore, we pre-processed the raw comment data as follows.

(1) Data denoising. Remove interfering information from the original comments, including duplicate comments, advertisements, comments that are irrelevant to the research, and meaningless words and symbols.

(2) The stopword dictionary is added. In order to improve the efficiency of word separation, a total of 1550 deactivated words were aggregated from the Chinese deactivation table of HIT and the Chinese deactivation table of Baidu [49].

(3) A customized word segmentation dictionary is constructed. Considering the complexity of online content, some particular words could not be identified in the word separation process. For example, the specialized words of rice types such as “long-grain rice” and “pearl rice”; the emotional words from evaluation such as “not good” and “don’t like”; and the e-commerce words such as “home delivery” and “logistics speed”. In order to improve the accuracy of word separation, we developed a custom dictionary through manual supervision, and the specific process is as follows: ① obtain the subject words with high word frequency in the product titles and the high-frequency words expressed by consumers; ② add the above words to the custom dictionary and carry out word separation; ③ according to the word frequency statistics of word separation, add the words with unreasonable word separation to the custom dictionary through manual checking, a total of 30 words (Table 2).

In the end, we obtained 77,429 valid reviews, of which 38,656 and 38,772 were for green-labelled rice and conventional rice, respectively.

### 3.3. Topic Modeling with LDA

This study uses the LDA model for thematic clustering of product reviews to discover the focus and attitudes of consumers towards green-labelled rice and conventional rice. The LDA is an unsupervised Bayesian probabilistic model for discovering the hidden semantics of textual data and contains a three-layer structure of words, topics, and documents [50]. The model is not only applicable to short Chinese texts but also has good reliability [51,52].

By training the model, the optimal number of topics can be found based on the topic confusion, and lower confusion values represent a better model fit [53]. However, the simplicity and interpretability of the textual content should be taken into account when selecting the number of topics [50]. By setting the different numbers of topics for the experiment, we obtained (num_topics, perplexity) = {⋯(9, 49,891.04), (10, 49,671.81), (11, 50,148.18)⋯}. When num_topics = 10 we get the minimum value of perplexity and therefore, the number of topics is determined as 10 in this research. 

### 3.4. Sentiment Analysis

This study uses SnowNLP, which is more established in the Chinese language domain, to perform sentiment analysis on product reviews [54]. The model is based on a machine learning Bayesian algorithm to train and predict the data, normalizing the sentiment score of each text to be between 0 and 1. The results of the model indicate the probability of the sentiment, with close to 1 being positive and close to 0 being negative [55]. The original training data of this model are different from the text data of this study. Therefore, in order to improve the accuracy of the sentiment calculation, the model needs to be retrained.

(1) We conducted a random sampling based on 10% of the number of valid comments. A total of 7743 comments were sampled to build the corpus.

(2) We invited two PhD level researches in food science and customer relationship management to individually annotate each comment in the corpus with positive and negative sentiment polarity within five working days. Comments with consistent annotation results were formally admitted into the corpus. For comments with inconsistent results, we invited a professor of management to perform a secondary annotation, taking the majority (2:1) as the final sentiment polarity according to the annotation results and eliminating the controversial comments. We obtained 7564 validly annotated comments in the end.

(3) In total, 80% of the valid annotated comments were used as the training set and 20% as the test set. The Sentiment module of SnowNLP was called for training and testing, and the original model was compared with the trained model. The results showed that the accuracy of the original model was 80.37%, the precision was 79.38%, and the recall rate was 79.28%; the accuracy of the trained model was 88.71%, the precision was 93.82%, and the recall rate was 91.08%. Therefore, the sentiment analysis model established in this study can effectively handle review texts in the food domain.

(4) The sentiment value (between 0 and 1) was calculated for the remaining 90% of valid comments using the retrained model. Sentiment values ≥ 0.6 were taken as positive sentiment and ≤ 0.4 as negative sentiment and comments with insignificant sentiment between 0.4 and 0.6 were excluded [56].

### 3.5. Statistical Tests

(1) We used SPSS to conduct a one-way ANOVA to determine whether there was a significant difference between the topics of green-labelled rice and conventional rice by comparing the mean weights of each topic.

(2) We used independent sample T-Tests to assess which topics differed significantly in mean weights. When the sample size is large, the significance level should be set at a lower level [57], and therefore based on $\alpha =0.05\times \sqrt{100/N}$ [58], we set the significance level at 0.0005.

(3) We calculated the absolute effect size of Cohen’s d by dividing the mean difference by the combined standard deviation [57]. Referring to the extended Monte Carlo study [59], the effect sizes were interpreted as follows: trivial (0 ≤ d < 0.2), small (0.2 ≤ d < 0.5), medium (0.5 ≤ d < 0.8), and large (0.8 ≤ d ≤ 1) [60].

## 4. Results

### 4.1. Consumer Concerns

**Figure 3:**
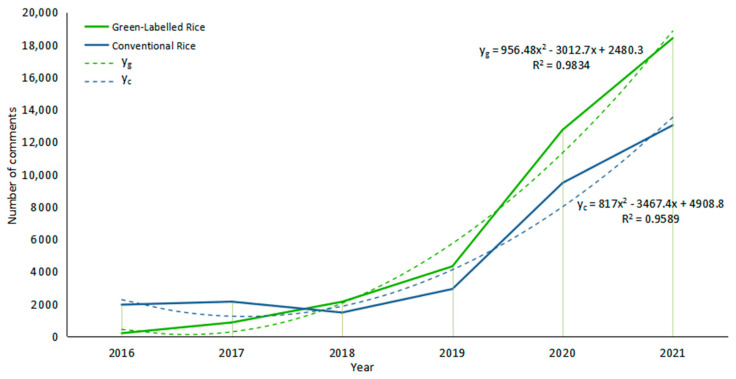
Level of attention on green-labelled rice and conventional rice.

We counted the number of consumer comments on green-labelled rice and conventional rice between January 2016 and April 2022. Figure 3 shows the fitted curves and the curves in the level of consumer attention to green-labelled rice and conventional rice. These two curves show the nearly same changing trends in consumer attention. The R^2^ of the two types of rice converges to 1, indicating a good fit. Overall, consumer attention in both types of rice has increased year after year. The introduction of national food safety policies between 2017 and 2018 has led consumers to focus on safer and more environmentally friendly green foods [61], with consumer concern in green-labelled rice overtaking conventional rice for the first time between 2017 and 2018, and consumer concern for green-labelled rice has proliferated since then. The estimated trend of the fitted curve shows that in the future, consumers will pay more attention to green-labelled rice than conventional rice.

Based on the LDA model, we extracted ten meaningful and relevant topics. These topics cover a range of issues, such as consumers’ perception of product features, the experience of the purchase process, and subjective evaluation of the product. We provided a name for each topic based on the high-frequency feature words extracted, as shown in Table 3. They are logistics speed, origin, taste and flavour, appearance characteristics, price, aroma, product packaging, impurity content, quality evaluation, and production date. The intensity of topics is obtained by calculating the probability distribution of documents representing users’ degree of attention regarding a topic [62]. Furthermore, the probabilities of each document belonging to different topics are the topics to calculate.

The extracted topics are potential influence factors that consumers were concerned about when purchasing rice [17], and they are based on the sensory experience of consumers experiencing the food. The human-food interaction is a multi-module experience in which consumers rarely separate individual senses independently [63]. When confronted with food stimuli, people perceive and integrate information from all senses through unconscious neurophysiological processes [28]. In the case of rice, this sensory information includes visual (appearance characteristics, product packaging, and impurity content), olfactory (aroma), and gustatory (taste and flavour) sensations. Appearance characteristics, impurity content, aroma, taste, and flavour reflect the quality of rice to a certain extent, while product packaging is a concern of consumers about the integrity of packaging and food safety during food transportation. At the same time, the essential attributes of rice, such as origin, price, and production date, are also factors of concern to consumers. In addition, for the e-commerce platform, as a channel for consumers to shop, logistics speed is the most important factor for consumers. Its transportation efficiency and service quality will also have a certain degree of influence on consumers’ experience of buying green-labelled rice.

### 4.2. Differences in Consumer Attitudes

**Figure 4:**
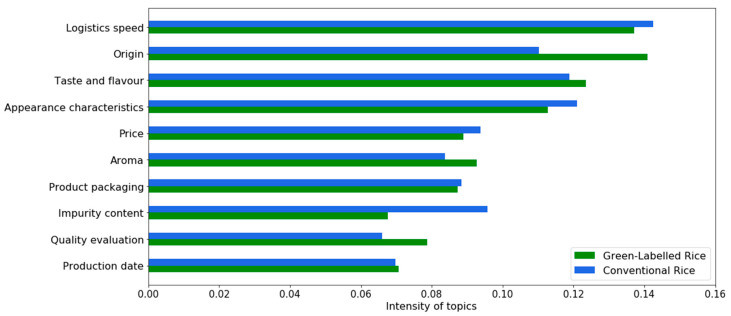
Comparison of topic weights for green-labelled rice and conventional rice.

Based on the LDA model, we portrayed the weight distribution of green-labelled rice and conventional rice on different topics (Figure 4). For green-labelled rice, origin, aroma, taste, and flavour are most important to consumers. For conventional rice, appearance characteristics, taste, and flavour are most important to consumers. In addition, the speed of logistics in e-shopping experience is of great concern to consumers of both types of rice.

Consumer attitudes towards the two types of rice differed under the same topic. Based on the topic weights, we conducted a one-way ANOVA and T-Test and used Cohen’s d Mean to indicate the magnitude of the differences (Table 4). Prior to this, we performed a normality analysis of the data. Due to the large sample size, we chose a K-S test. The results showed that the *p*-value > α = 0.0005, which indicated that the data were approximately normally distributed and could be subjected to subsequent statistical analysis. The results showed significant differences in consumers’ attitudes towards green-labelled rice and conventional rice across eight topics (intensity of topics = 0.8424), except for the two topics, which are the production date and the product packaging (intensity of topics = 0.1576).

According to the T-Test results, consumers who buy green-labelled rice are more concerned with origin, taste and flavour, aroma, and quality evaluation than conventional rice. Consumers who buy conventional rice are more concerned with logistics speed, appearance characteristics, price, and impurity content than green-labelled rice. Furthermore, according to Cohen’s d Mean value and Effect Size results, there is a trivial (d = 0.1) difference in consumer concerns about logistics speed, taste and flavour, appearance characteristics and price between green-labelled rice and conventional rice. However, the difference between concerns is less than 0.2 standard deviations, and the difference is insignificant and not meaningful [60,64]. In addition, there is a small (d = 0.2) difference in consumer concerns about the origin, aroma, impurity content, and quality evaluation.

Consumers who buy green-labelled rice are more aware of the origin and pay more attention to the quality of the rice. Consumers who are concerned about food safety are also usually concerned about origin information [16], as origin will match quality ratings [65] and the origin information on product packaging will influence consumer perceptions of product quality [66]. The green label means that the environment of origin meets environmental standards, so the quality and safety of green-labelled rice are more assured.

The aroma is a more distinctive feature of green-labelled rice, and this is what consumers look for. Green-labelled rice has higher culinary quality than conventional rice [14], and its overall quality and taste are better [65]. However, the results showed that the difference in consumer attitudes towards taste and flavour between the two types of rice was trivial (d = 0.1). The sense of smell affects consumers’ enjoyment and perception of food [67], and the difference in consumer attitudes towards rice aroma between the two types of rice is also relatively large (d = 0.2).

Foods’ overall liking and purchase intent are influenced by visual inputs [68]. Consumers of conventional rice were more concerned about impurities in rice (d = 0.2). Combining the characteristic words extracted from the impurity content topic, such as: “worm”, “mouldy”, and “yellowing”, indicates that significantly more problems with impurities occur in conventional rice than in green-labelled rice, which also means that there is still more room for improvement in quality control of conventional rice.

It is worth noting that price is an essential consideration for consumers when purchasing goods. The price of green-labelled rice is also generally higher than conventional rice. Previous research [21] has suggested that consumers who purchase green-labelled rice are not sensitive to price. However, we found a trivial (d = 0.1) difference in consumer attitudes towards price between the two types of rice, such that this difference was not practically significant. It shows that consumers who buy green-labelled rice are equally sensitive to price.

### 4.3. Differences in Consumer Sentiment

Food-elicited emotion is increasingly becoming critical for product differentiation [69]. Based on the sentiment values calculated by the SnowNLP model, we counted the number of positive and negative comments on each topic for both types of rice. As shown in Table 5, except for the topic of impurity content, the number of positive comments on each of the remaining nine topics for green-labelled rice was more significant than the number of negative comments. In total, 82.65% of consumers had positive sentiments towards green-labelled rice, 14.82% had negative sentiments, and 2.53% had little sentiment, indicating that consumers were relatively more satisfied with green-labelled rice. The non-parametric test results show that consumers have higher positive emotions about the logistics speed, origin, taste and flavour, and aroma of green-labelled rice, and more negative emotions about price, product packaging, impurity content and production date. In contrast, they had a more negative sentiment towards the impurity content of the rice and product packaging. In comparison, 63.62% of consumers had positive sentiments towards conventional rice, 33.50% had negative sentiments, and 2.87% had little sentiments. The results of the comparison of the topics of positive and negative emotions show that the number of positive emotions is higher in logistics speed, origin, appearance characteristics, and aroma. Whereas, the negative sentiment towards conventional rice mainly focused on rice quality, which shows that conventional rice has an apparent disadvantage in terms of quality.

The results of the T-test (Table 6) show a significant difference in the sentiment values between the two types of rice on 10 topics and that the probability of positive sentiment is greater for green-labelled rice than for conventional rice. In particular, in terms of product packaging, the difference in sentiment between the two types of rice is significant (d = 0.8), with consumers having an overall negative attitude towards the product packaging of conventional rice (mean (CR) = 0.3283 ≤ 0.4). In terms of rice impurity content, consumers have a low mean positive sentiment towards both types of rice, with both being negative. Consumers who purchase conventional rice have a higher probability of negative sentiment towards impurity content (1 − Mean (CR) = 1 − 0.1394 = 0.8606). In addition, the difference in the mean values of consumer sentiment towards the two types of rice in terms of speed of logistics, origin, and appearance characteristics is small, and both have a high probability of positive sentiment.

Further, we explored the specific reasons for each sentiment by analysing the frequency of words in comments of green-labelled rice and conventional rice [70].

**Figure 5:**
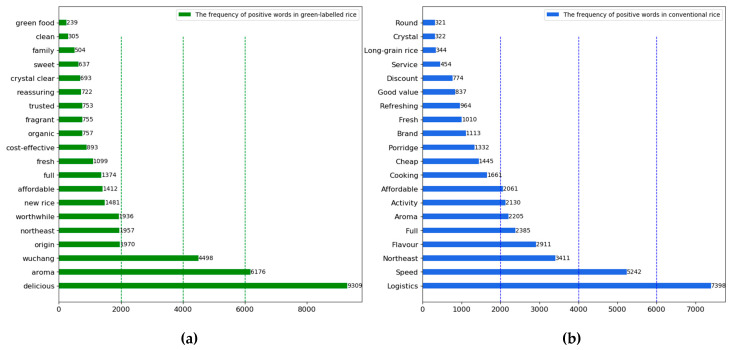
(**a**) Frequency of positive emotion-related words for green-labelled rice; (**b**) frequency of positive emotion-related words for conventional rice.

As shown in Figure 5a, the positive words of green-labelled rice mainly include “delicious”, “Wuchang”, “worthwhile”, “new rice”, “full”, “ fragrant”, “reassuring”, “family”, and “green food”. This shows that consumers have a positive attitude towards the quality, taste, and safety of green-labelled rice. As shown in Figure 5b, the positive terms of conventional rice include “logistics”, “speed”, “flavor”, “affordable”, “porridge”, “fresh”, “discount”, “service”, and “round”. This shows that consumers are more concerned about the value of consumption and service experience and have a positive attitude towards the cost-performance ratio of conventional rice. In particular, there is more positive feedback from consumers on the logistics speed and appearance characteristics of traditional rice.

**Figure 6:**
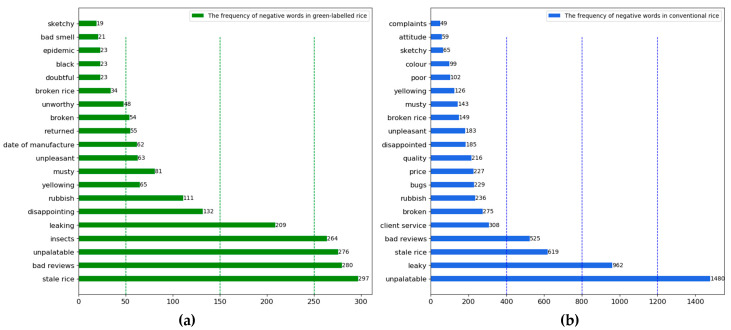
(**a**) Frequency of negative emotion-related words for green-labelled rice; (**b**) frequency of negative emotion-related words for conventional rice.

As shown in Figure 6a, the negative terms of green-labelled rice mainly include “stale rice”, “insects”, “leaking”, “disappointing”, “musty”, “broken”, “unworthy”, and “doubtful”. This indicates that consumers’ frustration with green-labelled rice is mainly reflected in distrust of the green food label and broken packaging. As shown in Figure 6b, the negative terms of conventional rice include “unpalatable”, “leaky”, “stale rice”, “broken”, “rubbish”, “bugs”, “unpleasant”, and “sketchy”. It shows that poor quality and broken packaging are the main reasons that consumers have negative sentiment, also, long storage time and excessive impurities impact consumer sentiment.

## 5. Discussion

Firstly, the pandemic is essential in raising consumer concerns about green-labelled rice. Public health incidents have increased consumer awareness of food safety, and health scares have prompted consumers to turn to healthier foods perceived as more natural [3]. Driven by egoism, consumers are more willing to purchase green food with quality assurance for their health [6,71]. Meanwhile, the pandemic has also triggered a sense of environmental concern and responsibility among consumers, leading to a greater awareness of personal social responsibility [24]. Environmental awareness has become a significant incentive for environmentally friendly food consumption [72]. Driven by altruism, consumers perceive the purchase of green food as an environmentally friendly act that demonstrates personal ethics and thus, holds positive attitudes towards green-labelled food. Consumers with high levels of altruistic environmental values are more likely to purchase green products [73]. This study supports Qi et al.’s [5] conclusion that consumers’ ethical attitudes influence the willingness to purchase green-labelled food. In addition, the strong government support for environmental protection and green consumption under the COVID-19 pandemic are driving factors for consumer interest in green label rice [74].

Secondly, price is an essential factor that consumers consider when making purchase decisions [75,76]. Stolz et al. [21] indicated that consumers who prefer green-labelled foods are not price-sensitive, and those who are price-sensitive prefer conventional products. Baudry et al. [7] also concluded that consumers who buy green-labelled foods are less concerned about price. However, we found that consumers who buy green-labelled rice are more concerned about price, and price-sensitive consumers also buy green-labelled rice. On the one hand, people’s income constraints or lower income expectations due to the pandemic could lead to spending less on green food [77] and caring about the price. Several studies have also confirmed that household income and expenditure affect consumers’ consumption of green foods [8,78]. In addition, the level of health and safety concerns motivates consumers to pay a premium for green foods, even when constrained by income [79]. On the other hand, the anonymous commenting mechanism on e-commerce platforms does not merely directly facilitate the expression of opinions by usually reticent consumers [80], it also facilitates the expression of genuine opinions and attitudes towards the purchased product [80,81] for effectively providing consumers with emotional safety [44]. Thus, authentic and massive consumer reviews bring objective consumer insights and reflect rational consumer behaviour during the COVID-19 pandemic.

Finally, mistrust of the green label is the leading cause of negative sentiment. On the one hand, although most consumers have a positive attitude toward green-labelled food, some remain skeptical of the safety, nutritional, and environmental quality characteristics due to the lack of proper and objective understanding of the green labels [18]. On the other hand, the presence of a few counterfeit products and low-quality green-labelled rice in the market has led to a bias in consumer expectations, triggering skepticism towards green-labelled rice, including distrust of third-party labelling verification [26]. Lack of label trust reduces consumer expectations of the benefits of green-labelled foods and has been a barrier to developing the green-labelled food market [82]. In addition, several studies have demonstrated that green food label certification has a significant impact on consumer attitudes toward green foods [83,84]. Yin et al. [85] found that the trust of consumers with high-risk perceptions in food safety has been reduced to a very low level, which also negatively affects their trust in Chinese certification labels. Wang et al. [86] also concluded that consumer trust in green labels and certification organizations significantly affects consumers’ willingness to purchase green labelled foods. This was also verified in our study from the sentiment analysis of consumers’ online reviews. Therefore, the government needs to promote knowledge about green labelling and strengthen the regulation of the green food market.

## 6. Conclusions

Understanding consumers’ perceptions and emotions towards green-labelled food is of genuine value for developing the green food industry and promoting green consumption, but also of strategic significance for building a low-carbon society and achieving green sustainable development. This study overcomes the limitations of traditional research in terms of data acquisition. It uses product reviews on e-commerce platforms to explore consumers’ different concerns and emotional tendencies toward green-labelled rice and conventional rice. The results show that consumers’ concerns about green-labelled rice have shown a significant upward trend since the COVID-19 out-break; consumers are more concerned about the origin, aroma, and taste of green-labelled rice than conventional rice; consumers of conventional rice are more concerned about the cost-performance ratio, while consumers of green-labelled rice are also price-sensitive; and that most consumers have a positive attitude towards green-labelled rice, while mistrust of green labels and broken packaging during logistics are the main causes of negative emotions.

### 6.1. Theoretical Contributions

Firstly, we applied the methods of text mining, sentiment analysis, and statistical tests to study consumer perceptions and sentiments towards green-labelled rice and conventional rice by collecting online reviews from the e-commerce platform. By using these methods, we can obtain more realistic and objective information, which is a big improvement compared with the previous literature that adopted interviews or questionnaires to survey. This approach suggests that review data from e-commerce platforms are useful for research in the field of green food, providing a new perspective for the study of public perceptions and emotions about green food, and contributing to the study of consumer behaviour in the field of green food.

Secondly, this study found that label mistrust was the leading cause of negative consumer sentiment towards green-labelled rice based on consumer review data, as consumers’ distrust of the green label directly led to questions about the quality of the product. It also further validates that the issue of label mistrust is critical in discouraging consumers from purchasing green-labelled foods [77].

Thirdly, price is an important influencing factor for consumers when purchasing goods. This study found that consumers who purchase green-labelled rice are also consistently concerned about price, in contrast to Stolz et al.’s [21] finding that consumers who purchase green-labelled rice are not price-sensitive. In addition, price-sensitive consumers also purchase green-labelled rice. In the context of the pandemic, increased concern about food safety and environmental health has directly contributed to greater consumer acceptance of green-labelled rice.

### 6.2. Implications for Practice

Firstly, to improve consumers’ trust in green labelling, the government should strictly supervise the origin of rice that meets the national environmental quality standards for green food and strengthen the governance of online and offline markets. Furthermore, the government should promote a unified green product certification and labelling system, while actively spreading and popularising knowledge of green food labelling.

Secondly, as the integrity of food packaging is a considerable and important factor for logistics [43], online retailers in the green food industry should reinforce the packaging quality of green-labelled rice to reduce the problem of packaging breakage during the shipping and courier process. At the same time, they should work closely with courier companies to improve transportation efficiency and provide optimal delivery solutions for consumers in different and remote regions.

### 6.3. Limitations and Future Research Directions

Due to the limitations of publicly crawling the product review on JD.com, we could not obtain the statistical characteristics of users who posted reviews, such as age, gender, education level, and income. In future research, assuming factors of consumers’ statistical characteristics as control variables, questionnaires and empirical analysis can further explore the differences in consumers’ perceptions and emotional characteristics of green-labelled rice and conventional rice under different sample dimensions. In addition, the level of consumers’ perception and trust in green labels is a crucial factor influencing consumers’ purchase intentions and decisions. However, existing research has not identified the psychological mechanisms. Therefore, we will adopt the social value theory and social trust theory to explain the mechanism of consumers’ trust in green labels.

## Figures and Tables

**Table 1:** Summary of Literature in the Field of Green-Labelled Rice.

Author	Method	Sample size	Conclusion
Qi & Ploeger [5]	Interview	28	The COVID-19 pandemic has increased consumers’ willingness to buy green food. The high price of green food, the problem of unavailability, the problem of mistrust, and limited knowledge are the main factors that trigger the IBG (Intentional Behavioural Gap).
Tong et al. [24]	Questionnaire	622	Subjective environmental knowledge and concerns about food quality have a significant impact on consumers’ willingness to purchase green-labelled rice, and individual socio-demographic characteristics affect consumers’ choice of green-labelled rice, including age, education, health status, and income.
My et al. [41]	Experiment	199	Consumers willing to pay premiums for sustainably produced rice are more health-conscious; have better knowledge and greater trust in food quality certification for rice; and tend to be more environmentally conscious. Enhancing consumers’ understanding and trust in food quality certification can help increase consumers’ acceptance of sustainably produced rice.
Anang et al. [42]	Interview	100	The most preferred attributes of rice are taste, cooking quality, cooking time, and aroma, and consumers are willing to pay higher premiums for the aroma and origin of rice. In contrast, the least preferred attributes are price, impurities, and product origin.
Hao et al. [43]	Text mining	25,000	Package integrity, delivery timeliness, door-to-door delivery, and service responsiveness are the most important logistical factors for consumers when purchasing rice and produce.
Hu et al. [14]	Content analysis	142,158	Green-labelled rice outperformed conventional rice in terms of appearance and cooking quality. However, in terms of protein content, there was no obvious difference between green-labelled rice and conventional rice.

**Table 2:** Custom dictionaries.

No.	Word (Chinese)	Word (English)	No.	Word (Chinese)	Word (English)
1	不好吃	Not good	16	送货上门	Home delivery
2	不香	Not fragrant	17	物流速度	Logistics speed
3	不喜欢	Don’t like	18	发货速度	Shipping speed
4	不划算	Not a good deal	19	京东快递	JDL express
5	米香	Rice fragrant	20	真空包装	Vacuum packed
6	新米	New rice	21	包装破损	Packaging broken
7	陈米	Stale rice	22	漏气	Leaking
8	长粒香	Long-grain rice	23	原产地	Origin
9	珍珠米	Pearl rice	24	绿色食品	Green food
10	旧米	Old rice	25	生产日期	Production date
11	五常大米	Wuchang rice	26	煮粥	Cooked porridge
12	颗粒均匀	Uniform grains	27	降价	Price reduced
13	颗粒饱满	Full of grains	28	性价比	Value for money
14	晶莹剔透	Crystal clear	29	发霉	Mouldy
15	软糯	Soft and sticky	30	国家地理标志	National geographical indication

**Table 3:** Topic Identification and Sorting of Intensity.

No.	Topic Identification	Intensity of Topic	Feature Words
1	Logistics speed	0.139834305	Logistics, Jingdong, speed, express, soon, service, home delivery, satisfaction, service attitude, epidemic
2	Origin	0.12553394	Northeast, Wuchang, ecological, Heilongjiang, South, Hubei, origin, trust, quality, Jingshan
3	Taste and flavour	0.121185015	Taste, flavour, tasty, delicious, soft, fluffy, sweet, fragrant, delicate, palatable
4	Appearance characteristics	0.115346825	Rice, full, grainy, crystal clear, clean, evenly grained, fresh, colour, translucent, broken rice
5	Price	0.08884901	Price, activity, cheap, value for money, discount, bargain, affordable, support, supermarket, guarantee
6	Aroma	0.088206845	Aroma, smell, fragrant, rice, delicious, worth, trust, brand, fragrant rice, aromatic
7	Product packaging	0.08736981	Vacuum packed, leaky, outer packaging, broken, epidemic, tight, shipping, complete, sealed, fine, intact, sturdy
8	Impurity content	0.08170846	Impurities, insects, rubbish, stale rice, lousy review, disappointment, broken rice, mouldy, yellowing, white spots
9	Quality evaluation	0.07233044	Quality, satisfied, loved, great, joyous, recommended, poor, new rice, affordable, five stars
10	Production date	0.070206845	Rice, fresh, date, taste, date of production, colour, old rice, month, new rice, local

**Table 4:** Comparison of Green-Labelled Rice (GLR) and Conventional Rice (CR).

No.	Topic	ANOVA (F-Value)	T-Test Result	Cohen’s d Mean	Effect Size
1	Logistics speed	13.114 *	*GLR < CR	0.1	Trivial
2	Origin	561.437 *	*GLR > CR	0.2	Small
3	Taste and flavour	11.983 *	*GLR > CR	0.1	Trivial
4	Appearance characteristics	113.309 *	*GLR < CR	0.1	Trivial
5	Price	95.328 *	*GLR < CR	0.1	Trivial
6	Aroma	78.356 *	*GLR > CR	0.2	Small
7	Product packaging	0.005	NS	NS	NS
8	Impurity content	776.172 *	*GLR < CR	0.2	Small
9	Quality evaluation	211.099 *	*GLR > CR	0.2	Small
10	Production date	1.040	NS	NS	NS

Not significant (NS): adjusted *p*-value > 0.0005, significant (*): adjusted *p*-value ≤ 0.0005.

**Table 5:** Distribution of the number of positive and negative comments for green-labelled rice and conventional rice and comparison of positive (Pos) and negative (Neg) topics.

No.	Topic	Green-Labelled Rice			Conventional Rice		
		Positive	Negative	Pos vs. Neg	Positive	Negative	Pos vs. Neg
1	Logistics speed	5296	448	*Pos > Neg	5122	1040	*Pos > Neg
2	Origin	5247	338	*Pos > Neg	3616	594	*Pos > Neg
3	Taste and flavour	4365	549	*Pos > Neg	2986	1394	*Pos < Neg
4	Appearance characteristics	3581	455	NS	3928	981	*Pos > Neg
5	Price	2068	486	*Pos < Neg	1691	1262	*Pos < Neg
6	Aroma	2740	156	*Pos > Neg	1841	370	*Pos > Neg
7	Product packaging	1843	901	*Pos < Neg	869	1940	*Pos < Neg
8	Impurity content	559	1253	*Pos < Neg	376	2965	*Pos < Neg
9	Quality evaluation	1884	237	NS	1004	473	*Pos < Neg
10	Production date	1215	341	*Pos < Neg	735	655	*Pos < Neg
	Total	28,798	5164		22,168	11,674	

Not significant (NS): adjusted *p*-value > 0.0005, significant (*): adjusted *p*-value ≤ 0.0005.

**Table 6:** Comparison of positive emotion probability between green-labelled rice (GLR) and conventional rice (CR).

No.	Topic	T-Test Result			Cohen’s d Mean	Effect Size
		Mean (GLR)	Mean (CR)	GLR vs. CR		
1	Logistics speed	0.9074	0.8188	*GLR > CR	0.3	Small
2	Origin	0.9123	0.8414	*GLR > CR	0.3	Small
3	Taste and flavour	0.8623	0.6704	*GLR > CR	0.5	Medium
4	Appearance characteristics	0.8685	0.7923	*GLR > CR	0.2	Small
5	Price	0.7887	0.5690	*GLR > CR	0.5	Medium
6	Aroma	0.9257	0.8175	*GLR > CR	0.4	Small
7	Product packaging	0.6494	0.3283	*GLR > CR	0.8	Large
8	Impurity content	0.3149	0.1394	*GLR > CR	0.5	Medium
9	Quality evaluation	0.8658	0.6709	*GLR > CR	0.5	Medium
10	Production date	0.7576	0.5307	*GLR > CR	0.6	Medium

Not significant (NS): adjusted *p*-value > 0.0005, significant (*): adjusted *p*-value ≤ 0.0005.

## Data Availability

The data are available from the corresponding author.
